# Optimization and stabilization of gold nanoparticles by using herbal plant extract with microwave heating

**DOI:** 10.1186/s40580-014-0012-8

**Published:** 2014-04-11

**Authors:** Akbar Yasmin, Kumaraswamy Ramesh, Shanmugam Rajeshkumar

**Affiliations:** Department of Biochemistry, Adhiparasakthi College of Arts and Science, Kalavai, 632506 Vellore District, TN India

**Keywords:** Gold nanoparticles, Green synthesis, Microwave, Stability, Optimization

## Abstract

In this study, we have synthesized the gold nanoparticles by using *Hibiscus rosa-sinensis*, a medicinal plant. The gold nanoparticles were synthesized rapidly by the involvement of microwave heating. By changing of plant extract concentration, gold solution concentration, microwave heating time and power of microwave heating the optimized condition was identified. The surface Plasmon resonance found at 520 nm confirmed the gold nanoparticles synthesis. The spherical sized nanoparticles in the size range of 16–30 nm were confirmed by Transmission Electron Microscope (TEM). The stability of the nanoparticles is very well proved in the invitro stability tests. The biochemical like alkaloids and flavonoids play a vital role in the nanoparticles synthesis was identified using the Fourier Transform Infrared Spectroscopy (FTIR). Combining the phytochemical and microwave heating, the rapid synthesis of gold nanoparticles is the novel process for the medically applicable gold nanoparticles production.

## Background

Nanotechnology is mainly concerned with the synthesis of nanoparticles of variable sizes, shapes, chemical compositions and controlled dispersity and their potential use for biomedical applications [1]. Although chemical and physical methods may successfully produce pure, well defined nanoparticles, these are quite expensive and potentially dangerous to the environment.

As an alternative to toxic and expensive physical methods for nanoparticles fabrication, using microorganisms, plants and algae will help a lot to synthesize the materials in the nano range and in addition, the toxicity of the by-product would be lesser than the other synthetic methods [2–4]. Many of the scientists are strongly supporting that there will not be any release of toxic substances during the nanoparticles synthesis with the help of green materials. The cause is chemicals which will be used in nanoparticles synthesis will get degraded by the enzymatic substances which are produced by the microbes during the time of growth. Plants also by trapping the bio-chemical materials with in their parts use the same as nutritive materials for metabolic processes of their own [5]. Using the biological organisms such as micro organisms [6], plant extract or plant biomass could be an alternative to chemical and physical methods for the production of nanoparticles [7,8].

Stabilization of gold nanoparticles using phyto-synthesis and microwave heating techniques is an emerging area in the field of advanced nanoparticles synthesis. Several plants and plant products have been successfully used for efficient and rapid extracellular synthesis of silver and gold nanoparticles. Leaf extracts of *Coleus aromaticus* [9], *Garcinia mangostana* [10], Barbated skullcup herb extract [11], *Magnolia kobus* and *Diopyros kaki* leaf extracts [12], *Rosa hybrid* petal extract [13], *Nyctanthes arbortristis* ethanolic flower extract [14], *Cassia fistula* leaf [15], *Lippia citriodora* (Lemon verbena) leaves extract [16] have also been used for gold nanoparticles synthesis. Using plants and plant materials for synthesis of metal nanoparticles could be advantageous over other environmentally benevolent biological processes by eliminating the elaborate process of maintaining bacterial and fungal cultures. It can also be easily scaled up for large scale production of beneficial nanoparticles.

A well known member of the family *Malvaceae*, *Hibiscus rosa-sinensis* grows as an important herbaceous plant. The bio-chemical constituents of the plants are Taraxery acetate, β-sitosterol, campesterol, stigmasterol, cholesterol, erogosterol, lipids, critic, tartaric and oxalic acids, fructose, glucose, sucrose, falvonoids and flavonoid glycosides [17], Hibiscentin, cyaniding and cyanin glucosides are alkanes. This plant has lot of applications like anti-infectious, anthelmintic, anti-inflammatory, diuretic, antipyretic [18]. The young leaves and flowers are used in inducing abortion and as a cure for headache. The plant has many hidden medical benefits [19].

In this study we synthesized the optimized gold nanoparticles by using medicinal plant extract *Hibiscus rosa-sinensis.* For the optimized production of gold nanoparticles, five different concentrations of *Hibiscus rosa-sinensis* leaf extract was added with gold chloride solution in different microwave heating voltage and time intervals. After the synthesis, the nanoparticles were confirmed by UV–vis spectrophotometry, Transmission Electron Microscopy and the stability of nanoparticles also measured by UV–vis spectroscopy.

## Methods

### Materials

The *Hibiscus* leaves for analysis were collected from Vellore District, South India. The chlororauric acid (HAuCl_4_), cysteine, Sodium chloride and sodium phosphate were purchased from Hi Media, Mumbai.

### Green synthesis of optimized gold nanoparticles

2, 4, 6, 8 and 10 g of *Hibiscus rosa-sinensis* leaves cut into appropriate size was taken to in 100 ml wide neck borosil conical flask and washed several times with deionised water. 100 ml deionised water was added to the flask containing freshly cut *Hibiscus rosa-sinensis* leaves exposed to microwave heating for 3 min. The resultant crude extract was filtered with Whatman filter paper No. 40 and used for synthesis process. 1 mM of gold chloride solution was taken for the gold nanoparticles synthesis.

Optimization of gold nanoparticles synthesis was done by changing the parameters like Plants extracts concentration, temperature (Power of microwave heating) and Microwave heating time. The details of the optimization parameters are given in Tables 1, 2, 3, 4 and 5.

**Table 1:** **Preparation of gold nanoparticles by 2% (2 ml) leaf extracts concentration**

**Volume of gold solution 1 mM (μl)**	**Microwave heating time (sec)**	**Power of microwave heating (W)**
300	30	140
300	60	140
300	90	140
300	30	280
300	60	280
300	90	280
300	30	420
300	60	420
300	90	420

**Table 2:** **Preparation of gold nanoparticles by 4% (4 ml) leaf extracts concentration**

**Volume of gold solution 0.1 M (μl)**	**Microwave heating time (sec)**	**Power of microwave heating (W)**
300	30	140
300	60	140
300	90	140
300	30	280
300	60	280
300	90	280
300	30	420
300	60	420
300	90	420

**Table 3:** **Preparation of gold nanoparticles by 6% (6 ml) leaf extracts concentration**

**Volume of gold solution 0.1 M (μl)**	**Microwave heating time (in sec)**	**Power of microwave heating (W)**
300	30	140
300	60	140
300	90	140
300	30	280
300	60	280
300	90	280
300	30	420
300	60	420
300	90	420

**Table 4:** **Preparation of gold nanoparticles by 8% (8 ml) leaf extracts concentration**

**Volume of gold solution 0.1 M (μl)**	**Microwave heating time (sec)**	**Power of microwave heating (W)**
300	30	140
300	60	140
300	90	140
300	30	280
300	60	280
300	90	280
300	30	420
300	60	420
300	90	420

**Table 5:** **Preparation of gold nanoparticles by 10% (10 ml) leaf extracts concentration**

**Volume of gold solution 0.1 M (μl)**	**Microwave heating time (Sec)**	**Power of microwave heating (W)**
300	30	140
300	60	140
300	90	140
300	30	280
300	60	280
300	90	280
300	30	420
300	60	420
300	90	420

### Characterization of gold nanoparticles

Preliminary characterization of gold nanoparticles was carried out using UV–vis spectroscopy (Tecan plate reader-infinite m200 model). Gold nanoparticle powder sample was prepared by centrifuging the synthesized gold nanoparticle solution at 10,000 rpm for 15 min for Fourier transformed infrared radiation spectroscopy measurement. The solid residue layer which contains gold nanoparticles was dispersed in sterile deionised water three times to remove the attached biological impurities. The pure residue was then dried in an oven overnight at 65°C. The obtained powder was subjected to FT-IR measurement carried out on a Perkin Elmer spectrum-one instrument at a resolution of 4 cm^−1^ in KBr pellets. Transmission electron microscopy measurement of synthesized gold nanoparticles using *Hibiscus rosa-sinensis* leaf extract were carried out on Techni-20 Philips transmission electron microscope operated at 80 kev. TEM sample were prepared by the dispersion of 2–3 drops of *Hibiscus rosa-sinensis* stabilized gold nanoparticle solution on a copper grid and dried at room temperature after the removal of excess solution with filter paper.

### *In-vitro* stability studies of synthesized gold nanoparticle using *Hibiscus rosa-sinensis* leaves

*In-vitro* stability study of optimized *Hibiscus rosa-sinensis* stabilized gold nanoparticle was tested in the presence of 10% NaCl, 0.2 M Cysteine, PBS 6, PBS 7.4 and PBS 8. Typically, 1 ml of gold nanoparticle solution was added to 24 well plates containing 0.5 ml of 10% NaCl, 0.2 M Cysteine, PBS (pH 6, pH 7.4 and pH 8) solution, respectively and incubated for 30 min. The stability and the identity of gold nanoparticle were measured by recording UV absorbance after 30 min. The biomedically important plant mediated gold nanoparticles were autoclaved and it was analysed by UV–vis spectrophotometer.

## Results and discussion

### Synthesis of gold nanoparticles

**Figure 1:**
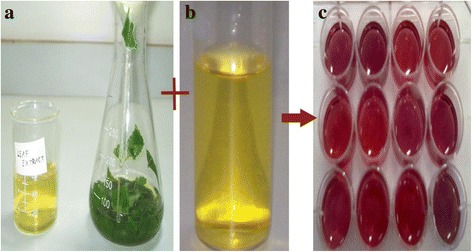
**Visual observations (a) Plant leaf extract (b) gold chloride solution and (c) formation of gold nanoparticles.**

Formation of gold nanoparticles by reduction of aqueous metal ions during exposure of microwave radiation to the plant extract may be easily followed by UV–vis spectroscopy. It is well known that the gold nanoparticles exhibit ruby red colour (Figure 1) in water. The appearance of ruby red colour is the characteristics of gold nanoparticles it clearly indicates formation of gold nanoparticles. This colour formation is due to the surface Plasmon vibration of the metal nanoparticles [20]. In case of gold nanoparticles, the narrow surface Plasmon resonance band occurred at 520 nm as shown in figures.

Reduction of gold ions in to gold nanoparticles is the time taking process, in early studies on synthesis of gold nanoparticles using microorganism required 2 to 120 hrs [21–23,6], but our plant extract stabilized microwave radiation technique studies shows rapid formation of gold nanoparticles within a minute. Using of microwave radiation to nanoparticles synthesis has benefit that it provides uniform heating of aqueous solution and prevent the particles from aggregation [24].

### Production of optimized gold nanoparticles

**Figure 2:**
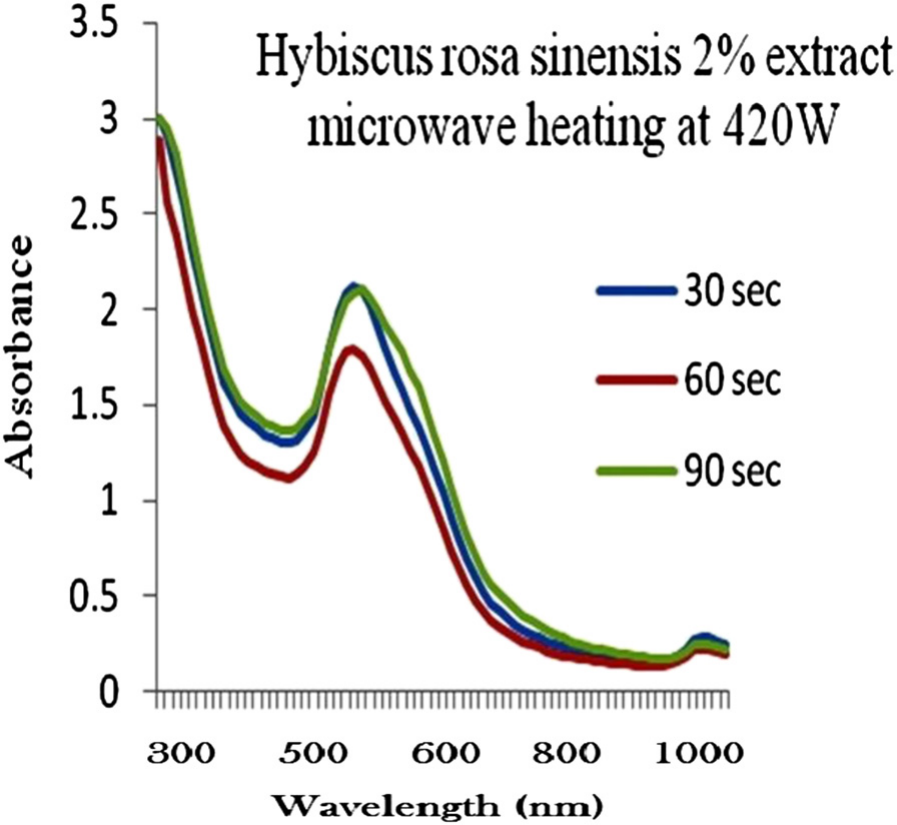
**2% of Plant extracts and microwave heating at three different temperature.**

**Figure 3:**
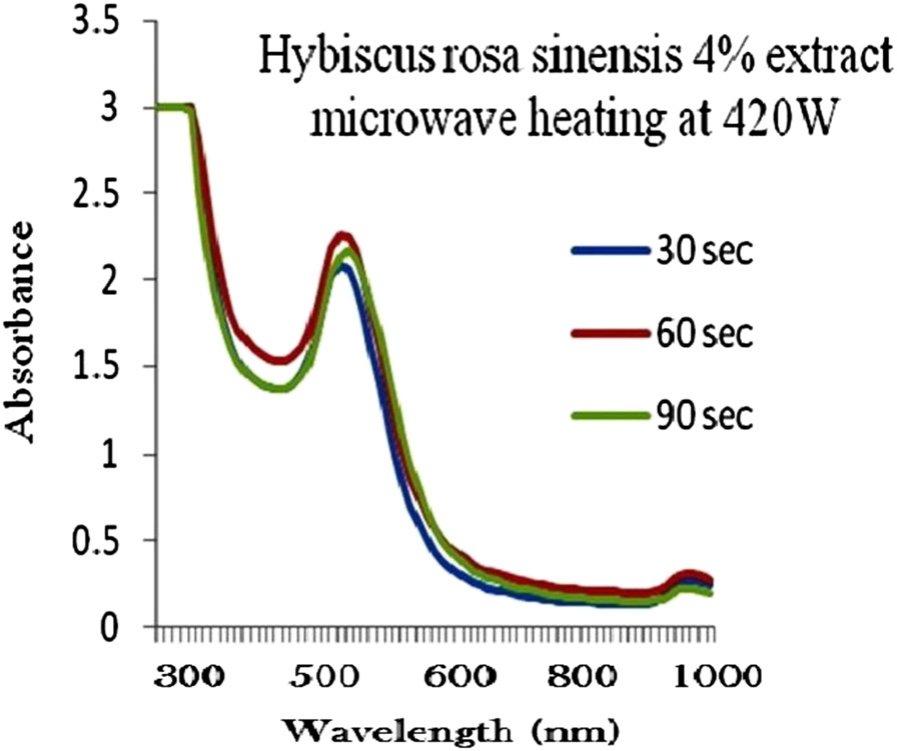
**4% of Plant extracts and microwave heating at three different temperatures.**

**Figure 4:**
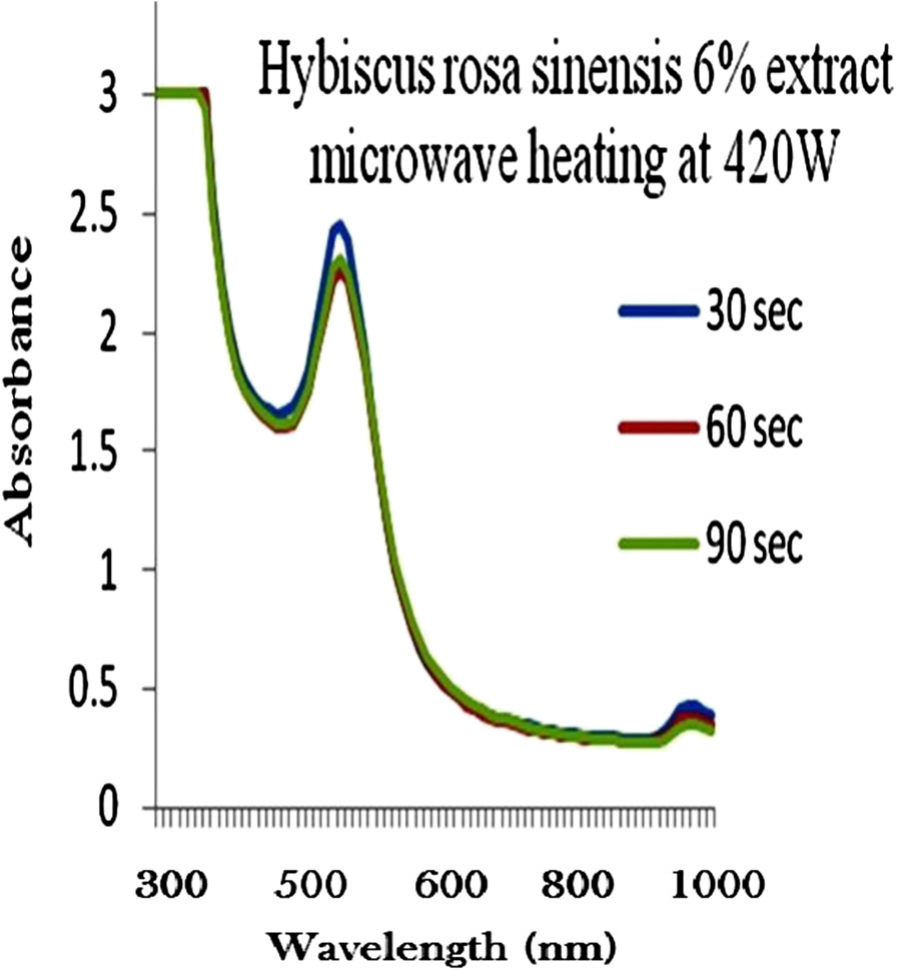
**6% of Plant extracts and microwave heating at three different temperature.**

**Figure 5:**
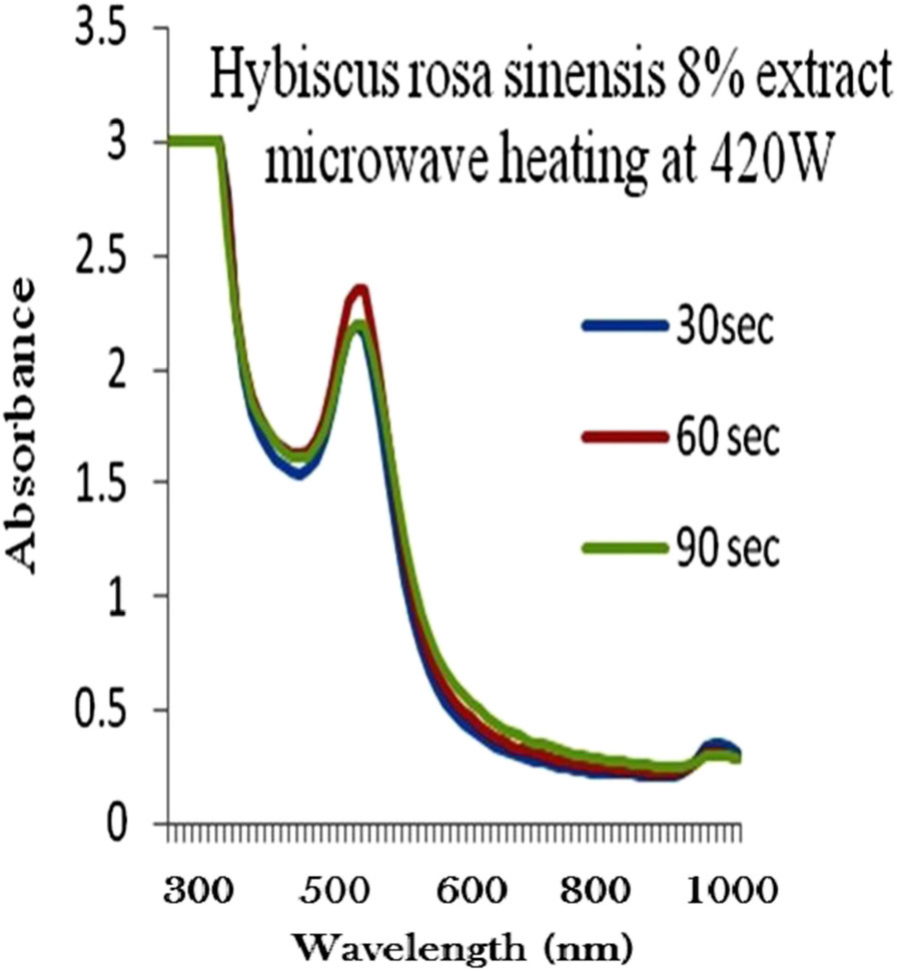
**8% of Plant extracts and microwave heating at three different temperature.**

**Figure 6:**
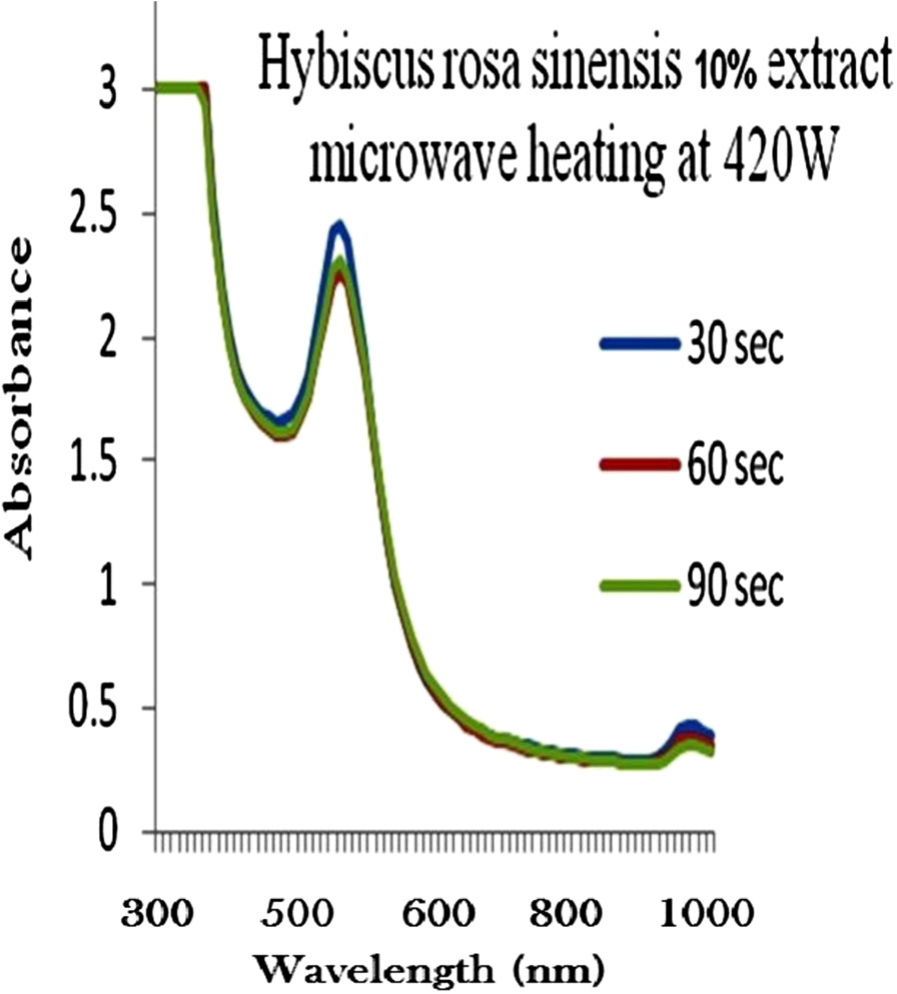
**10% of Plant extracts and microwave heating at three different temperature.**

The process of optimization of gold nanoparticles was carried out by keeping the gold concentration and volume of plant extract solution as constant and changing the microwave radiation power time and plant extract concentration. Figures 2, 3, 4, 5 and 6 represents UV-visible absorption spectra of *Hibiscus rosa-sinensis* stabilized gold nanoparticle formed when changing the process variables like concentration of plants extract from lower to higher concentration (2–10%), microwave radiation power from 140–420 W and time of exposure from 30–90 sec. From the absorption spectra it can be seen that plant extract concentration plays an important role in controlling the size and shape of nanoparticles. A single and narrow absorbance band was observed at 520 nm which is characteristics for the formation of small nanoparticles which was confirmed by TEM result. While increasing the leaf extract concentration up to 8%, the absorbance intensity steadily increased as a function of time of reaction. At 10% leaf extract concentration treated with gold ion solution, the absorbance decreased due to the bioavailability of functional groups in leaf extract for gold ions leads to the competition between leaf extract and metal ions for reduction process (Figure 6).

Among the variables, plant extract 8% concentration microwave radiation power 420 W and microwave exposure time in 90 sec, was ideal for gold nanoparticles preparation, because it shows (Figure 5) gold surface resonance occur at ~520 nm and steadily increased in intensity as a function microwave power and time of reaction without any shift in peak wavelength. There is no change in peak wavelength suggest that particles are monodispersed in the aqueous solution without aggregation [4].

### Stability testing

**Figure 7:**
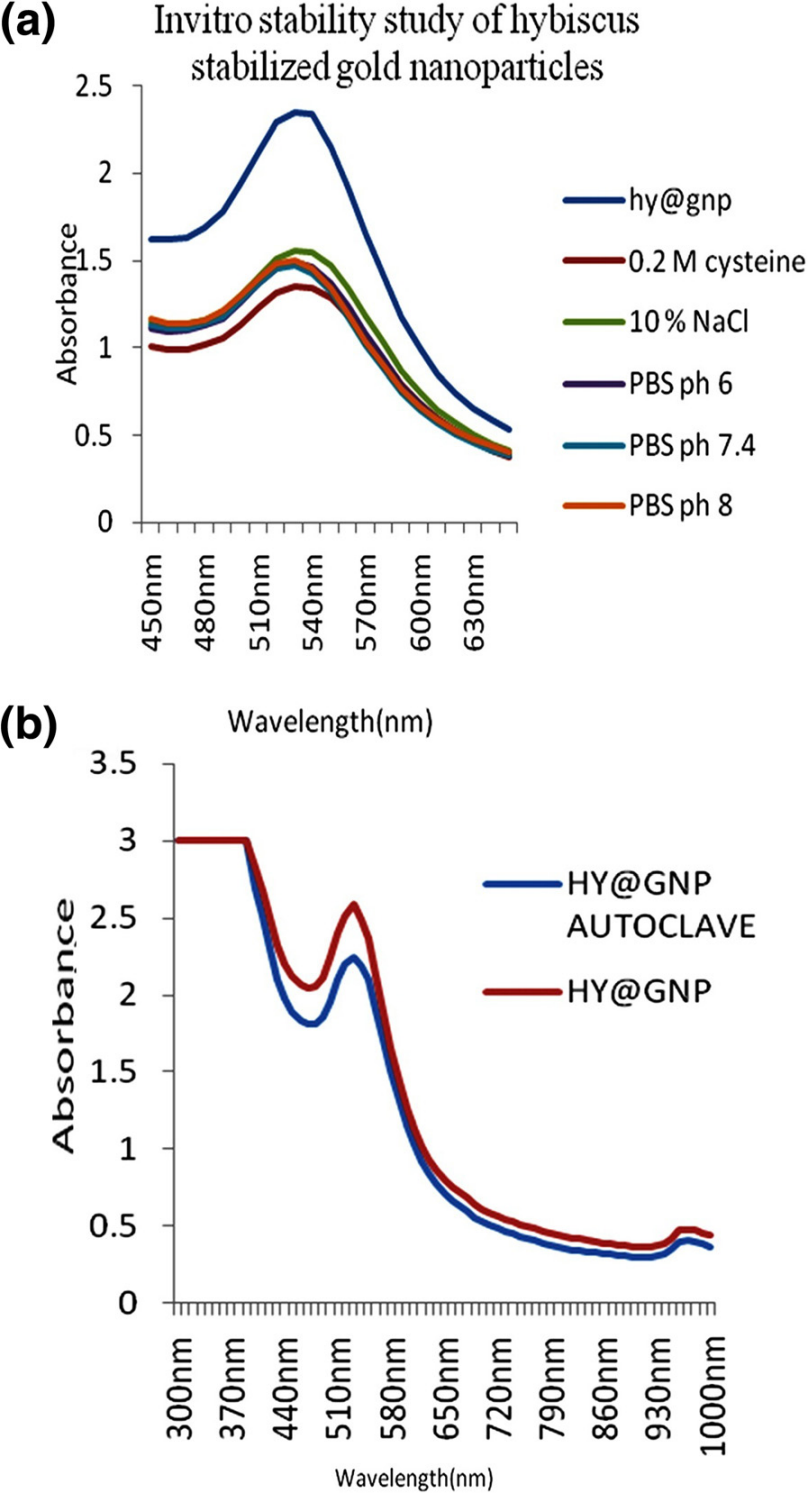
**Stability analysis of gold nanoparticles (a) by various parameters and (b) absorbance before and after autoclaving.**

The stability of *Hibiscus rosa-sinensis* stabilized gold nanoparticles was evaluated by monitoring the plasmon λ_max_ in 0.2 M cysteine, 10% NaCl, phosphate buffer solution at pH 6, 7.4 and 8. The plasmon wavelength in all above shows shift of ~1 – 8 nm. Our result from the *in-vitro* stability studies has confirmed that gold nanoparticles were stable in biological fluids at physiological pH (Figure 7).

Gold nanoparticles used varies biomedical application before going to *in-vivo* models. Sterilization is very important process to complete destruction of all organism including bacteria, spores, virus.

Several sterilization methods can be used, including physical methods such as autoclaving and UV irradiation, which comprise moist heat and drug heat, respectively and chemical treatment such as using hydrogen peroxide gas plasma, ethylene oxide and chemical vapour, which include both gaseous and liquid solutions.

Sterilization remains a critical step for the *in-vivo* use of gold nanoparticles, and the effects of sterilization on the integrity of the physiochemical properties of gold nanoparticles used to be investigated. Hence, gold nanoparticles sterilized by autoclave at 120°C for 15 min before and after sterilization of gold nanoparticles shows similar surface plasmon resonance peak ~520 nm. It indicates that the gold nanoparticles size did not changed by sterilization. The particles are thus stabilized by the capping agent like flavonoids and water soluble compounds present in the leaf extract [25].

### TEM

**Figure 8:**
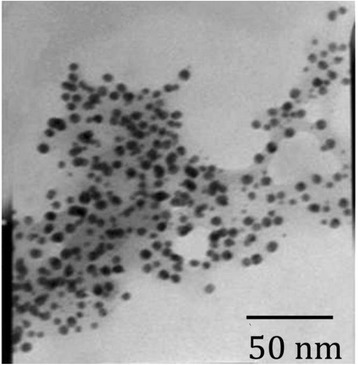
**TEM image of gold nanoparticle synthesized from** ***Hibiscus rosa-sinensis.***

A TEM study reveals the size and shape of nanoparticles. The shape of gold nanoparticles prepared in this study is spherical with size in the range of 16 – 30 nm (Figure 8). Some nanoparticles are seen as aggregated. In the surface of the nanoparticles some light colour of materials found that may be the stabilizing agent responsible for the nanoparticles synthesis [26]. The separation between nanoparticles was observed from TEM image due to the presence of capping agent which is characteristics to well dispersed nanoparticles formation in the optimized conditions.

### FT-IR

**Figure 9:**
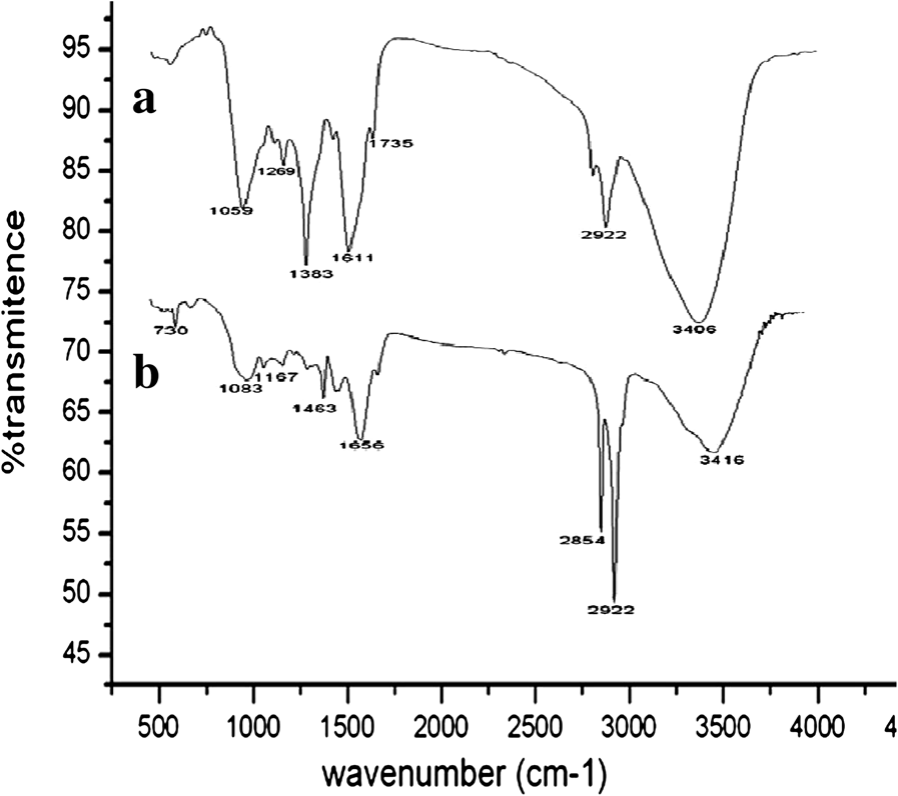
**FT-IR spectrum of (a)** ***Hibiscus rosa-sinensis*** **leaf extract and (b) gold nanoparticles.**

The plants are having a lot of bio-chemical molecules like stigmasterol, erogosterol, Hibiscentin and falvonoids play an important role in synthesis and stabilizing of gold nanoparticles [27,28]. These compounds may actively involve in the reduction of gold ions to gold nanoparticls was characterized by FTIR spectrum of leaf extract before and after reduction process. In the FTIR spectrum (Figure 9) of *Hibiscus rosa-sinensis* multiple peaks were observed between 735 and 1059 cmˉ^1^ representing various functional groups of the alkaloids and flavonoids and other bimolecular functions. There were no amides groups present in the synthesized gold nanoparticles powder. After the bioreduction of HAuCl_4_ by the extract of *Hibiscus rosa-sinensis* considerable shifts were observed in the IR spectrum of *Hibiscus rosa-sinensis* gold nanoparticle. Major shifts observed were the peak at 1611 cm^−1^ shifted to 1656 cm^−1^ and the peak at 1383 cm^−1^ disappeared. The medium and sharp peak at 1059 shifted to 1083 cm^−1^ and changed into small blunt peak. The peaks observed show that alkaloids or flavonoids would have adsorbed on the metal surface by the interaction with carbonyl group/keto groups. Similarly the functional groups in the *Hibiscus* extract could have been oxidized during the reduction of HAuCl_4_ to gold nanoparticles. The shifted peaks in the plants extracts and nanoparticles clearly indicates the nanoparticles formation by the plant extracts of *Hibiscus rosa-sinensis* acts as reducing and stabilizing agent [29,30].

## Conclusions

By using green chemistry, the gold nanoparticles were synthesized by microwave radiation method which is a rapid method and gives excellent reproducibility when compared to other synthetic processes. Using plants for nanoparticle synthesis can be advantageous over other biological process by eliminating the elaborate process of maintaining cell cultures. The gold nanoparticles have showing high stability rate in ‘*in-vitro*’ conditions and can be applicable for biological applications. Thus the field of gold nanoparticle biosynthesis is leading to avenues in material science, chemistry, nanobiotechnology and biotechnology. The single-step green process has shown to be effective for the generation and stabilization of non-toxic gold nanoparticles for different applications in a myriad of diagnostic purpose and therapeutic applications. *Hibiscus rosa-sinensis* mediated eco-friendly synthesized gold nanoparticles have excellent stability under different chemicals treatment and sterilization. Due to this stability characterization of gold nanoparticles could be used for biomedical and sensor applications. Thus the design and development of ‘green’ gold nanoparticles can be safely produced, stored and shipped worldwide.

## References

[1] Rajeshkumar S, Kannan C, Annadurai G (2012). Synthesis and characterization of antimicrobial silver nanoparticles using marine brown seaweed *Padina tetrastromatica*. Drug Invent. Today.

[2] Malarkodi C, Rajeshkumar S, Paulkumar K, Gnanajobitha G, Vanaja M, Annadurai G (2013). Eco-friendly synthesis and characterization of gold nanoparticles using *Klebsiella pneumonia*. J. Nanostruct. Chem..

[3] Vanaja M, Gnanajobitha G, Paulkumar K, Rajeshkumar S, Malarkodi C, Annadurai G (2013). Phytosynthesis of silver nanoparticles by *Cissus quadrangularis* - influence of physico-chemical factors. J. Nanostruct. Chem..

[4] Rajeshkumar S, Malarkodi C, Vanaja M, Gnanajobitha G, Paulkumar K, Kannan C, Annadurai G (2013). Antibacterial activity of algae mediated synthesis of gold nanoparticles from *Turbinaria conoides*. Der. Pharma. Chemica..

[5] Gnanajobitha G, Paulkumar K, Vanaja M, Rajeshkumar S, Malarkodi C, Annadurai G, Kannan C (2013). Fruit mediated synthesis of silver nanoparticles using *Vitis vinifera* and evaluation of their antimicrobial efficacy. J. Nanostruct. Chem..

[6] Malarkodi C, Rajeshkumar S, Paulkumar K, Vanaja M, GnanaJobitha G, Annadurai G (2013). Bactericidal activity of bio mediated silver nanoparticles synthesized by *Serratia nematodiphila*. Drug Invent. Today.

[7] Vanaja M, Rajeshkumar S, Gnanajobitha G, Paulkumar K, Malarkodi C, Annadurai G (2013). Kinetic study on green synthesis of silver nanoparticles using *Coleus aromaticus* leaf extract. Adv. Appl. Sci. Res..

[8] Gnanajobitha G, Rajeshkumar S, Kannan C, Annadurai G (2013). Preparation and characterization of fruit-mediated silver nanoparticles using pomegranate extract and assessment of its antimicrobial activity. J. Environ. Nanotechnol..

[9] Vanaja M, Annadurai G (2012). *Coleus aromaticus* leaf extract mediated synthesis of silver nanoparticles and its bactericidal activity. Appl. Nanosci..

[10] Karthiga P, Soranam R, Annadurai G (2012). Alpha-mangostin, the major compound from *Garcinia mangostana* Linn. Responsible for synthesis of Ag nanoparticles: its characterization and evaluation studies. Res. J. Nanosci. Nanotechnol..

[11] Wang Y, He X, Wang K, Zhang X, Tan W (2009). Barbated skullcup herb extract-mediated biosynthesis of gold nanoparticles and its primary application in electrochemistry. Colloids Surf. B: Biointerfaces.

[12] Song JY, Jang HK, Kim BS (2009). Biological synthesis of gold nanoparticles using *Magnolia kobus* and *Diopyros kaki* leaf extracts. Process Biochem..

[13] Noruzi M, Zare D, Khoshnevisan K, Davoodi D (2011). Rapid green synthesis of gold nanoparticles using Rosa hybrid petal extract at room temperature. Spectrochim. Acta A.

[14] Das RK, Gogoi N, Bora U (2011). Green synthesis of gold nanoparticles using *Nyctanthes arbortristis* flower extract. Bioproc. Biosyst. Eng..

[15] Lin L, Wang W, Huang J, Li Q, Sun D, Yang X, Wang H, He N, Wang Y (2010). Nature factory of silver nanowires: plant mediated synthesis using broth of *Cassia fistula* leaf. Chem. Eng. J..

[16] Cruz D, Fale PL, Mourato A, Vaz PD, Luisa Serralheiro M, Lino ARL (2010). Preparation and physicochemical characterization of Ag nanoparticles biosynthesized by *Lippia citriodora* (Lemon Verbena), colloid. Surf. B Interf..

[17] Olagbende-Dada SO, Ezeobika FN, Duru FI (2007). Anabolic effect of Hibiscus *rosa-sinensis* Linn. Leaf extracts in immature albino male rats. Nig. Q. J. Hosp. Med.

[18] Sharma S, Sultana S (2004). Effect of *Hibiscus rosa-sinensis* extract on hyperproliferation and oxidative damage caused by benzoyl peroxide and ultraviolet radiations in mouse skin. Basic Clin. Pharmacol. Toxicol..

[19] Sachdewa A, Khemani LD (1999). A preliminary investigation of the possible hypoglycemic activity of *Hibiscus rosa-sinensis*. Biomed. Environ. Sci..

[20] Rajeshkumar S, Malarkodi C, Paulkumar K, Vanaja M, Gnanajobitha G, Kannan C, Annadurai G (2013). Seaweed mediated synthesis of gold nanoparticles using *Turbinaria conoides* and its characterization. J. Nanostruct. Chem..

[21] Rajeshkumar S, Kannan C, Annadurai G (2012). Green synthesis of silver nanoparticles using marine brown algae *Turbinaria conoides* and its antibacterial activity. Int. J. Pharm. Bio. Sci..

[22] Husseiny MI, Abd El-Aziz M, Badr Y, Mahmoud MA (2007). Biosynthesis of gold nanoparticles using *Pseudomonas aeruginosa*. Spectrochim. Acta A.

[23] Mukherjee P, Senapati S, Mandal D, Ahmad A, Khan MI, Kumar R, Sastry M (2002). Extracellular synthesis of gold nanoparticles by the fungus *Fusarium oxysporum*. Chembiochem.

[24] Saifuddin N, Wong CW, Yasumira AAN (2009). Rapid biosynthesis of silver nanoparticles using culture supernatant of bacteria with microwave irradiation. E-J. Chem..

[25] Philip D, Unni C, Aromal SA, Vidhu VK (2011). *Murraya Koenigii* leaf-assisted rapid green synthesis of silver and gold nanoparticles. Spectrochim. Acta A.

[26] Iosin M, Baldeck P, Astilean S (2010). Study of tryptophan assisted synthesis of gold nanoparticles by combining UV–Vis, fluorescence, and SERS spectroscopy. J. Nanopart. Res..

[27] Philip D, Unni C (2011). Extracellular biosynthesis of gold and silver nanoparticles using Krishna tulsi (*Ocimum sanctum*) leaf. Phys. E..

[28] Mohammed-Fayaz A, Girilal M, Venkatesan R, Kalaichelvan PT (2011). Biosynthesis of anisotropic gold nanoparticles using *Maduca longifolia* extract and their potential in infrared absorption. Colloid. Surf B: Biointerf..

[29] Philip D (2009). Biosynthesis of Au, Ag and Au–Ag nanoparticles using edible mushroom extract. Spectrochim. Acta A.

[30] Raghunandan D, Basavaraja S, Mahesh B, Balaji S, Manjunath SY, Venkataraman A (2009). Biosynthesis of stable polyshaped gold nanoparticles from microwave-exposed aqueous extracellular anti-malignant guava (*Psidium guajava*) leaf extract. Nano Biotechnol..

